# Computational Modeling as a Tool to Investigate PPI: From Drug Design to Tissue Engineering

**DOI:** 10.3389/fmolb.2021.681617

**Published:** 2021-05-20

**Authors:** Juan J. Perez, Roman A. Perez, Alberto Perez

**Affiliations:** ^1^ Department of Chemical Engineering, Universitat Politecnica de Catalunya, Barcelona, Spain; ^2^ Bioengineering Institute of Technology, Universitat Internacional de Catalunya, Sant Cugat, Spain; ^3^ The Quantum Theory Project, Department of Chemistry, University of Florida, Gainesville, FL, United States

**Keywords:** protein-protein interactions, linear peptide motifs, computational methods, epitopes, tissue engineering, p53, Bcl-2

## Abstract

Protein-protein interactions (PPIs) mediate a large number of important regulatory pathways. Their modulation represents an important strategy for discovering novel therapeutic agents. However, the features of PPI binding surfaces make the use of structure-based drug discovery methods very challenging. Among the diverse approaches used in the literature to tackle the problem, linear peptides have demonstrated to be a suitable methodology to discover PPI disruptors. Unfortunately, the poor pharmacokinetic properties of linear peptides prevent their direct use as drugs. However, they can be used as models to design enzyme resistant analogs including, cyclic peptides, peptide surrogates or peptidomimetics. Small molecules have a narrower set of targets they can bind to, but the screening technology based on virtual docking is robust and well tested, adding to the computational tools used to disrupt PPI. We review computational approaches used to understand and modulate PPI and highlight applications in a few case studies involved in physiological processes such as cell growth, apoptosis and intercellular communication.

## Introduction

Most proteins mediate complicated metabolic and signaling pathways through interaction with other proteins, either in the form of dimers or as components of larger complexes (Hunter, 2000; Stelzl et al., 2005). Some of these interactions are transient, while others are more permanent. It is estimated that there are approximately 650,000 types of specific protein-protein interactions (PPI) in a human cell (Stumpf et al., 2008) and as much as 40% of these are mediated by short peptide linear binding motifs (London et al., 2013). Actually, protein domains involved in PPIs often bind multiple peptides that share linear motifs—common sequence patterns—like for example, the canonical SH3 domain-binding PxxP motif (Mayer, 2001). These motifs are often embedded within locally unstructured protein regions but can also bind their partners as short, isolated peptides acting as PPIs inhibitors.

Mapping PPIs is key to understand a wide range of physiological processes such as cell growth, apoptosis and intercellular communication. In turn, anomalies in protein interaction networks including the concentration of a specific protein in the cell are associated with diseases such as cancer, infectious diseases, and neurodegenerative diseases (Gonzalez and Kann, 2012). Accordingly, a detailed understanding of the human interactome--the complex network of PPIs—(Luck et al., 2020) offers novel opportunities for therapeutical intervention (Milroy et al., 2014).

Designing different kinds of PPIs modulators, including inhibitors that arrest signaling by disrupting a specific PPI or enhancers that restore signaling through facilitation of a specific PPI is now a major goal in controlling cell processes and pathways. In addition, allosteric binders can provide a different type of PPI modulation, acting through the selective perturbation of a protein interaction with specific partners and modulating a signaling pathway accordingly (Cesa et al., 2015). Indeed, this procedure opens the possibility of designing two different disruptors acting on the same target that produce different cellular responses due to the way they alter local PPI networks. Conceptually, designing PPI disruptors is simpler than designing enhancers, since the latter require finding a short linker that brings the two proteins in close contact to each other, whereas the former require finding molecules that bind to any site of any of the two proteins to prevent their association.

Designing modulators of PPIs is challenging compared to ligands targeting enzymes or GPCRs, due to the specific features of protein-protein interfaces including a large and flat interfacial area (∼1500–3000 Å^2^), lacking in grooves or binding pockets (Wells and McClendon, 2007). Moreover, the PPI binding free energy is characterized by a large buried hydrophobic surface area, suggesting a large entropic contribution—although electrostatic complementarity of interacting protein surfaces is also important in PPIs. Consequently, the binding free energy is not correlated to the PPI surface area buried. For example, the complex of the tumor suppressor p53 protein to its negative regulator MDM2 described below is an example of a small contact surface area and high affinity (Borcherds et al., 2014), while the complex of the Bcl2-associated athanogene (Bag) and an eukaryotic chaperone 70-kDa heat shock protein (Hsp70) exhibits a high surface area, but a small binding free energy (Sondermann et al., 2001). Interestingly, PPI surfaces exhibit a reduced number of key contributors or “hot spots” to the binding free energy (<2 kcal/mol) that are usually found at the center of the interface. Tryptophan, arginine and tyrosine are the most frequent residues identified as “hot spots”, whereas other residues such as valine, lysine or serine rarely participate (Hu et al., 2000). The occurrence of key residues in PPIs was demonstrated for the first time in the seminal study of the complex of the human growth hormone (hGH) and the extracellular domain of its receptor (gGHbd) (Clackson and Wells, 1995). In order to understand the contribution of each residue to the PPI, the authors produced all the possible gGHbd mutants generated by substitution of each individual residue involved in the PPI surface area by alanine. The process permitted to show that 8 out of 31 side chains involved in the PPI surface area contributed about 85% to the binding free energy of the complex. Alanine scanning technique is currently carried out *in vivo* using phage display technology in a combinatorial fashion (Morrison and Weiss, 2001) and computational strategies to perform alanine scanning have also been developed (Kortemme et al., 2004).

**FIGURE 1:**
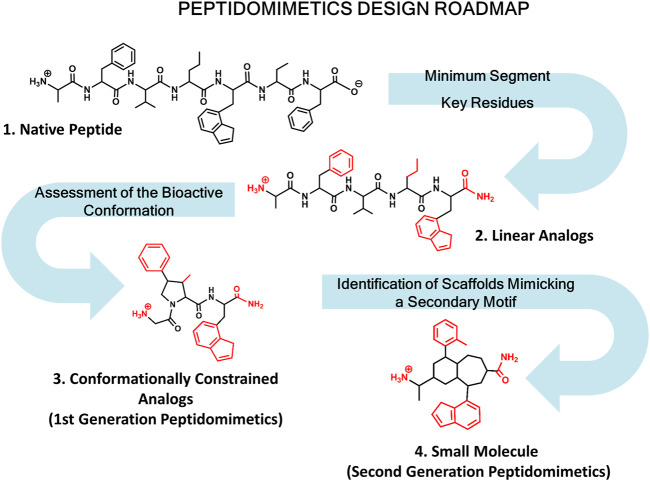
Roadmap for peptidomimetics design. Starting from the native peptide (1), first step regards establishing the shortest peptide fragment with activity, as well as identification of key residues involved in recognition (in red) (2). Next, using information about the secondary structure of the peptide in its bound conformation, proceed to design conformationally constrained analogs mimicking the bioactive conformation (3). These analogs represent the first generation peptidomimetics. Finally, identification of small molecule scaffolds that permit a correct spatial arrangement of relevant chemical groups (identified in step 2) to yield second generation peptidomimetics (4).

Diverse kinds of molecules from small molecules to antibodies have been used in the past as PPI disruptors (Gordo and Giralt, 2009). Among them, peptides have emerged as privileged molecules (Nevola and Giralt, 2015; Bruzzoni-Giovanelli et al., 2018). Analysis of protein complexes show that a large number of structures involve short linear peptide binding motifs and a globular protein domain. Interestingly, these peptide segments are responsible for most of the binding free energy (Petsalaki and Russell, 2008). Peptides are flexible and can adapt themselves to large surfaces, can be easily optimized and are safe and well tolerated. However, peptides exhibit limitations as drugs including the means of administration, poor pharmacokinetic profile and bioavailability. Despite their drawbacks to be used as drugs, peptides can be modified to produce peptidomimetics and peptide surrogates including cyclic peptides with an improved ADME profile. Peptidomimetics refers to small molecules that mimic key stereochemical features of the bioactive conformation of a target peptide (Perez et al., 2002; Akram et al., 2014; Perez, 2018; Santini and Zacharias, 2020; Tomasella et al., 2021). Designing peptidomimetics requires knowledge of key residues involved in peptide-protein interactions and structural features of the bioactive conformation. When the 3D structure of the complex is available, the process is simpler, however most of the times the bioactive conformation needs to be assessed using a combination of biophysical and computational methods together with the synthesis and biological evaluation of the molecules designed. In this case the roadmap normally followed for designing peptidomimetics is shown in Figure 1.

Below we describe the use of computational methods in conjunction of other biophysical and medicinal chemistry techniques to help to understand the features of peptide ligands, necessary to design PPI disruptors peptidomimetics and peptide surrogates and describe a few examples in drug discovery and tissue engineering.

## Structural Features of Protein-Peptide Interactions

The increased awareness on the role of peptide epitopes for mediating PPI has evidenced the need for a deeper structural understanding. Complexes are underrepresented in the PDB with respect to their biological prevalence, and experimental techniques often require expensive approaches (e.g., isotope labeling in NMR) to characterize peptide-protein interactions. Computational tools are a promising approach to bridge the gap. The goal of computational methods is to predict where and how a peptide would bind a protein receptor, distinguish which peptide sequences might be better binders, and help in the design process. Improvements in computational tools continue to push our ability to gain structural insights applicable to design principles.

Curated peptide-protein databases such as pepBDB (Wen et al., 2019), peptiDB (London et al., 2010) or pepBind (Das et al., 2013) compile a list of known protein-peptide systems found in the Protein Data Bank (PDB) (Berman et al., 2000). These curated databases are useful for the training, classification and understanding of peptide-protein interactions. Analyzing these databases helps to classify the types of interaction according to peptide structure, binding interface or degree of challenge for computational methods amongst other metrics. For example, Arkin and co-workers classified three types of peptide-protein binding (Arkin et al., 2014) according to the primary, secondary or tertiary structure they adopt. The primary structure binding motif is represented by short linear peptides such as those present in interactions between the extracellular matrix and membrane bound integrins (Ruoslahti, 1996). Secondary structure motifs involve the peptide adopting a determined secondary structure (e.g., alpha helix or beta strand) such as in the p53-MDM2 interaction (Kussie et al., 1996) or the BH3-Bcl-2 interaction (Adams and Cory, 1998), whereas tertiary structure represents discontinuous binding sites such as the XIAP-Smac interaction (Wu et al., 2000). A different classification for peptide-protein interactions is by looking at the protein-protein interface they are posed to inhibit (Yan et al., 2008). Peptides binding deep cavities are typically easier to model than those that interact with extended flat surfaces. Finally, one of the more challenging aspects is the degree of plasticity in the binding mode. This includes the conformational changes of the protein receptor as well as the ability to accommodate multiple binding conformations. This is especially characteristic of protein interaction hubs such as those involved in gene regulation (Aiyer et al., 2021). Many peptides are intrinsically disordered and fold upon binding, hence optimization of peptide design will often try to maximize interactions in the complex as well as the propensity of the peptide to adopt bound structures (e.g. through chemical staples or cyclization). Peptidomimetics are often a solution in which the peptide interactions are closely maintained while designing a molecule with a lower number of degrees of conformational freedom.

## Computational Methods Used to Study Protein-Peptide Interactions

Approaches such as docking and free energy perturbation are now routinely used in the drug discovery process for small molecules. Docking methodologies explore libraries of millions of virtual compounds in search for small molecule scaffolds or for drug repurposing efforts (Kitchen et al., 2004). Meanwhile, free energy perturbation methods are now able to calculate relative (and absolute) binding free energies with errors comparable to experiments (Wang et al., 2015). Despite the successes, they have well known limitations regarding the nature of the ligands (e.g., flexibility or charge) as well as the structural rearrangements needed in the protein receptor, which become important for peptide-protein systems.

Since 2001, the CAPRI (Critical Assessment of Predicted Interactions) (Janin et al., 2003) has been instrumental in assessing the developments in methods that predict macromolecular assemblies as well as scoring functions that can identify the best predictions. The focus has been in interactions between a protein and other proteins, nucleic acids and peptides–with an emphasis on protein-protein interactions. CAPRI is a blind prediction effort in the spirit of CASP (Critical Assessment of Structure Prediction) (Moult et al., 1995): research groups from around the world use their methods to predict atomic resolution 3D structures for targets provided by CAPRI, and to predict the ranking of models based on scoring functions. The event operates with strict time deadlines for each target. At the end of the event, assessors rank the predictions comparing them to the experimental structure (which is unknown to the community at the time of the predictions). Assessors are also blind to which group performed each prediction (double-blind experiment). These methods perform well for easy targets in which there are small changes in the proteins between their free and bound form. Performance decreases for both scoring and prediction methods for the “hard” targets in which binding requires conformational changes in the proteins with respect to their free form (Lensink et al., 2019).

The above limitations become relevant for the study of peptide-protein systems. Many peptides are intrinsically disordered in their free form, becoming structured during the binding process. Predicting bound conformations requires tackling this conformational flexibility and estimating the entropic contribution of adopting bound conformations to the free energy. Knowing the bound conformation of a protein-peptide system does not imply that other peptide sequences can adopt the same bound conformation. This binding plasticity is best exemplified by: 1) receptors that bind different peptide sequences in different conformations (Aiyer et al., 2021) and 2) peptide sequences that can adopt different structures when binding different receptors (Huart and Hupp, 2013). This amount of binding plasticity is a challenge for predicting peptide-protein interactions and for computing binding affinities.

Amongst the difficulties in predicting bound conformations for protein-ligand interactions is the mechanisms by which binding takes places. It is now widely accepted that binding happens through a combination of two main binding mechanisms, traditionally described as induced fit and conformational selection. The mechanism behind induced fit is that a ligand will bind in the active site and the protein/ligand system will undergo a conformational transition toward the bound conformation. In the conformational selection mechanism, the protein and ligand each have an ensemble of possible conformations available to them—and binding occurs through a specific conformation. Each particular system under study will have a different contribution from each mechanism. Thus, how much a system changes from the unbound/bound conformation is often used as a measure of the difficulty in predicting binding. The fly casting mechanism (Shoemaker et al., 2000; Sugase et al., 2007) has been coined to understand how intrinsically disordered domains can accelerate molecular recognition. This binding mechanism is often exhibited in peptide epitopes involved in PPIs. The resulting peptides are often intrinsically disordered as described in the above paragraph, resulting in a more challenging scenario than in small molecules, where now both the protein and peptide molecules can change conformations significantly from their free form.

### Docking-Based Approaches

An array of docking methodologies have emerged in the last few years, in response to a growing pharmacological interest for peptide-based drugs. Excellent reviews on such methods have been published (Ciemny et al., 2016; Porter et al., 2019; Aderinwale et al., 2020), along with studies providing benchmark sets for assessing current and future methods. Here we provide an overview of the general principles behind these approaches as well as efforts from the simulation community in predicting protein-peptide interactions.

Docking methods operate in different search modes depending on the known information about the system of interest. The ultimate goal is to recover the binding site and binding mode for the protein-peptide conformation. This problem can be broadly divided in two parts: the search problem and the scoring problem. The search problem is related to exploring the relative peptide-protein relative position and orientation, as well as the internal conformation of the protein and peptide. The scoring stage aims to identify the correctly bound structures amongst all predictions based on a function that relates docking structures to a score. Assessment of success is done on the top scoring poses.

For computational efficiency, docking methods reduce the search space in several ways, depending on the known information about the system. In a global search strategy, the peptide explores binding at all possible sites along the protein-receptor surface (Pierce et al., 2011; Kurcinski et al., 2015; Alam et al., 2017; de Vries et al., 2017; Porter et al., 2017; Xu et al., 2018; Zhou et al., 2018). Whereas in a local search approach, the binding region is limited based on prior knowledge, resulting in a more directed search (Jain, 2007; Antes 2010; Donsky and Wolfson, 2011; London et al., 2011; Trellet et al., 2013; Rentzsch and Renard, 2015; Lamiable et al., 2016; Antunes et al., 2017; Zhang and Sanner, 2019). Finally, template-based approaches forego this search by building flexibility on top of models extracted from structural databases based on protein and peptide alignments (Lee et al., 2015; Obarska-Kosinska et al., 2016).

The methods can be further divided based on how much conformational freedom is allowed for the protein receptor and the peptide. The protein receptor flexibility is a common problem to protein-protein and protein-small molecule docking (Andrusier et al., 2008; B-Rao et al., 2009). Approaches to model protein flexibility include the use of soft potentials (Fernández et al., 2002; Ferrari et al., 2004), explore rotameric states (Leach, 1994), using different protein receptor structures (Amaro et al., 2018; Falcon et al., 2019) or refining with molecular dynamics (Alonso et al., 2006). The challenges in modeling peptides arises from: 1) peptides can have different conformations in their free/bound states; 2) the same peptide sequence might bind different proteins in different conformations (Huart and Hupp, 2013), and 3) different peptide sequences can bind the same receptor in different conformations (Aiyer et al., 2021). Most docking methods use a flexible strategy for the peptide conformation. Rather than exhaustively sampling all possible conformations two approaches are typically taken: 1) based on sequence, and 2) based on representative peptide conformations. In the first approach the peptide sequence is used to either query the pdb for possible conformations of the peptide fragment (e.g., in proteins that contain that sequence) or predict secondary structure (Yan et al., 2016). The second approach uses several initial conformations generally adopted by peptides during binding (e.g., helix or extended) (see Weng et al., 2020).

Scoring functions have the task of identifying which poses are likely to be biologically relevant. Ideally these functions should reflect the underscoring binding affinities of different poses and compounds (Li et al., 2021). Their use dates back to the early days of docking methods to understand protein complexes (Kuntz et al., 1982). Scoring functions are typically divided in four types: 1) empirical fits, 2) knowledge based, 3) machine learning, and 4) first principles (Li et al., 2019; Li et al., 2021). One of the challenges in the adequate development of scoring functions is the ability to generate poses that include both good binders and bad binders. These has led to the development of decoy sets (Graves et al., 2005; Stein et al., 2021), often used as training sets for new functions. How these decoy sets are generated influences the corresponding scoring functions, often resulting in biases. Recent efforts aim at detecting and overcoming such biases (Morrone et al., 2020). Scoring functions that work for protein-protein or protein-small molecule systems are not always transferable to protein-peptide systems, resulting in the development of several specific peptide-protein scoring functions (Raveh et al., 2011; Kurcinski et al., 2015; Spiliotopoulos et al., 2016; Tao et al., 2020).

Despite the importance of blind studies (Lensink et al., 2007), there have been few protein-peptide targets in CAPRI in recent years (Weng et al., 2020). Benchmarking studies (Agrawal et al., 2019; Santos et al., 2020; Weng et al., 2020) and peptide protein datasets (London et al., 2010; Hauser and Windshügel, 2016) provide the community with the tools needed to improve docking and scoring methodologies. These benchmarks are typically divided into easy/medium/hard categories according to the conformational changes that the peptide has to undergo to bind (Trellet et al., 2013; Weng et al., 2020). A recent benchmark study using 14 different docking programs (Weng et al., 2020) shows that despite improvements in peptide-protein docking, predicting binding modes when large conformational changes are involved remains challenging.

### Free Energy-Based Approaches

We refer in this category to methods that produce ensembles obeying detailed balance, and using statistical mechanics to infer representative structures and free energies. Sampling is generally achieved through Monte Carlo (Metropolis et al., 1953; Hansmann and Okamoto, 1999) or Molecular Dynamics (MD) (McCammon et al., 1977) approaches using a force field to represent atomic interactions and capture the entropic contribution in the ensembles. In these approaches, a single point structure evaluation of the potential (given a force field) is not relevant to identify low free energy states—whereas scoring functions in docking or knowledge-based potentials are intended to evaluate single structures. Through the sampling of the free energy landscape these methods capture kinetics, mechanisms of action and binding affinities. The challenge in these methodologies is to sample timescales relevant to binding events. Simulations now routinely sample the microsecond timescale, but binding events typically require reaching the millisecond timescale. Despite improvements using specialized hardware (Pan et al., 2017), using brute force MD for binding remains computationally unfeasible. A common strategy is to use MD based approaches as the last stage of docking pipelines, leading to refined models. Recently, improvements in advanced sampling techniques and computer efficiency are opening new opportunities to study peptide-protein binding.

Free energy perturbation (FEP) methods are the golden standard for characterizing binding free energies of small molecules (relative or absolute) (Wang et al., 2015). FEP requires knowledge of the bound state and uses a path independent approach, generally combining alchemical transformations and restraints to evaluate the binding free energy change. These methods suffer when there are large changes in the scaffold of the molecule, the overall charge or when multiple binding modes have to be included (Gill et al., 2018; Ruiter and Oostenbrink, 2020; Wallraven et al., 2020). Thus, the successes of FEP for small molecules are not yet generally transferable to protein-protein or protein-peptide systems. Advanced sampling strategies are opening possibilities to study the peptide binding process and extract binding affinities, albeit at a greater computational expense.

Although there are many advanced sampling techniques, for the purpose of peptide binding we distinguish between those that capture kinetics and mechanisms of action and those that identify states and binding free energies. Advances in frameworks that combine multiple unbiased MD trajectories to recover kinetic and mechanistic properties such as Markov State Models (Noé et al., 2009; Bowman et al., 2010), weighted ensemble methods (Huber and Kim, 1996; Zhang et al., 2010) and milestoning (Faradjian and Elber, 2004; Votapka and Amaro, 2015) have been used to study several peptide-protein systems (Giorgino et al., 2012; Zwier et al., 2016; Paul et al., 2017; Zhou et al., 2017). These methods can be used to estimate on and off-rates, with off-rates being significantly harder to obtain due to the long timescales needed to observe unbinding events.

A second class of advanced sampling strategies combines known information (e.g., from experiments) and generalized ensemble methodologies (Sugita and Okamoto, 1999; Fukunishi et al., 2002) to identify bound conformations (Morrone et al., 2017a; Morrone et al., 2017b; Lang and Perez, 2021). These approaches are also used to obtain qualitative relative binding affinities from competitive binding simulations (Morrone et al., 2017a; Morrone et al., 2017b).

There are yet few instances of using these methodologies as well as progress towards more quantitative methodologies. For example, in frameworks that use multiple unbiased trajectories questions like how many simulations to start from each state, how to reweight them or how to visualize them in a space that allows interpretation of the ensembles is an area of active development.

### From Bound Conformations to Sequence Design

A final strategy used by the community is to identify mutations to a peptide sequence that will favor interaction with the protein through the use of fast approaches that rely on statistical or empirical potentials (Takano et al., 1999; Guerois et al., 2002) described in the docking section. Here, we typically have knowledge of a protein-protein interaction and the related structure, and use the binding epitope as a structural template. These methods are then used to mutate each residue in the peptide into different amino acids, using a fast scoring function to predict those mutations that lead to greater affinity (Delgado et al., 2019; Torres et al., 2019).

In what follows, we describe three specific examples of PPIs involved in diverse physiological process including cell growth, apoptosis or intercellular communication, together with a summarized description of the advances carried out for the development of peptide analogs, surrogates or peptidomimetics.

## The p53-mdm2/x interaction

P53 has been named the “guardian of the genome” for its tumor-suppressor activity. It is a protein interaction hub, predicted to be involved in over a thousand PPIs (Tan et al., 2019) through its different functional domains (May and May, 1999). In this section we will focus on the interaction between the p53 transactivation domain and the MDM2 protein (or its homologous MDMX) which marks p53 for degradation. Inhibition of the p53-MDM2/X interaction has been an important cancer target, since it liberates p53 to carry its tumor-suppressor activity. Despite the homology between MDM2 and MDMX, developing dual inhibitor drugs remains an active field of research, with several candidates in clinical trials.

**FIGURE 2:**
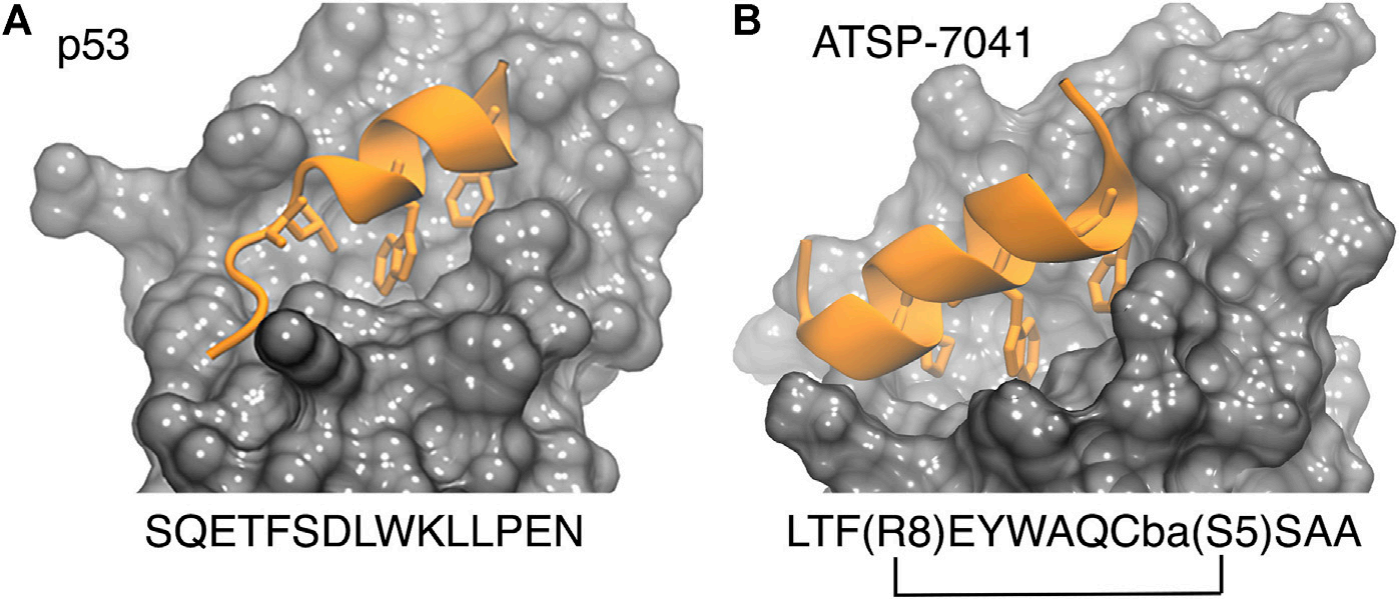
P53 (**left**, PDB 1ycr) and a stapled peptide (**right**, MELD prediction) binding MDM2. Three anchoring hydrophobic residues in the peptide as shown as sticks (orange). The cartoon representation shows the extended helix for the ATSP7041 stapled peptide.

The p53-MDM2 interaction involves a short intrinsically disordered epitope from the terminal transactivation domain of p53 binding as a helix to the N-terminal domain of MDM2 (Kussie et al., 1996). MDM2 has a deep hydrophobic cavity which anchors three residues from p53 (Phe^19^, Trp^23^ and Leu^26^; see PDB id 1YCR, see Figure 2), (Kussie et al., 1996). MDMX shares an 80% homology in the binding site with MDM2, resulting in p53 binding along the same binding mode (PDB id 3DAB) (Hu et al., 2006; Popowicz et al., 2008). Despite their similarity, MDMX presents a shallower binding site, which poses difficulties for developing binding inhibitors. Computational approaches have played a role in both the rational design of small molecules (Bowman et al., 2007) and peptides as potential drugs (Tan et al., 2016).

Studies based on MD and docking have tried to characterize the details of the p53-MDM2 interaction. Studies using MSM approaches are now yielding on-rates for p53-MDM2 close to experiment, while the off-rates remain challenging to estimate directly (Zwier et al., 2016; Zhou et al., 2017). These studies are also providing information about the binding mechanisms, predicting the helicity needed for a peptide to switch from an induced fit binding paradigm to one in which conformational selection dominates (Zhou et al., 2017). Some studies are also using the longer MDM2 construct, which includes a “lid” region that effectively reduces the amount of time the binding site is accessible for p53 binding. Molecular dynamics approaches yield detailed information on the effect of the lid disordered region on the binding energy surface of MDM2 and compares it to the case of p53 and several other small molecule drugs (Bueren-Calabuig and Michael, 2015). Recent flexible docking simulations of the p53 peptide starting from unbound conformations and including the disordered tails in MDM2 reported the best scoring structure to be 3.74 Å from the experimentally bound structure (Ciemny et al., 2018).

**FIGURE 3:**
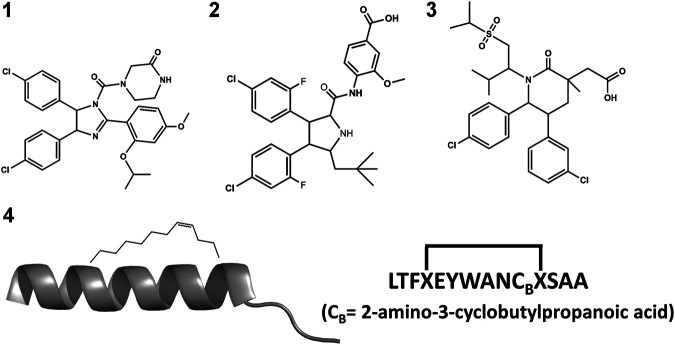
Chemical structures of nutlin-3a (1); idasanutlin (2); AMG-232 (3) and ATSP-7041 (4).

The small molecule drug Nutlin-3a (**1** in Figure 3) and its derivative, idasanutlin (**2** in Figure 3) now in pPhase III trial for relapsed/refractory acute myeloid leukemia were developed for its ability to bind an inhibit MDM2 (Vassilev et al., 2004; Vassilev, 2005; Ding et al., 2013). However, this family of compounds are not efficient against MDMX (Hu et al., 2006; Joseph et al., 2010)—similar trends have been observed in other compounds like AMG-232 (**3** in Figure 3), where binding to MDMX has significantly lower affinity than for MDM2. Furthermore, small molecules inhibitors of MDMX have not been successful in culture cells (Reed et al., 2010). Small molecule designs have tried to mimic the three hydrophobic residues found in the p53 binding epitope as a template for efficient inhibitor design (Gonzalez-Lopez de Turiso et al., 2013; Furet et al., 2016; Burgess et al., 2016) while reducing their toxicity. Several such designs are currently undergoing clinical trials (Burgess et al., 2016).

A different strategy for dual inhibition identifies peptide sequences based on the known binding motif. As a result, several linear peptides designs (Pazgier et al., 2009; Phan et al., 2010) with greater affinity than the original p53 peptide have been found. Peptide designs conserve the three hydrophobic residues that anchor in MDM2/X and make longer helices. Brownian Dynamics was used to investigate differences in binding kinetics for several peptide sequences (ElSawy et al., 2016). Despite the greater binding affinity to MDM2 and MDMX as mentioned above, linear peptides exhibited a poor ADME profile: they are easily degraded and hampered to cross barriers, limiting their use as drugs. They are however great starting points for peptidomimetic design. A different strategy uses non-standard amino acid backbones (Sang et al., 2020) to increase resistance to degradation while keeping the side-chains that allow strong interactions with the protein receptors. Several such peptides have advanced to clinical trials (Carvajal et al., 2018; Jiang and Zawacka-Pankau, 2020).

Stapled peptides represent an interesting alternative to overcome some limitations of linear peptides by easily crossing barriers, being resistant to degradation and adopting stable helical conformations that favor binding (Bernal et al., 2007; Chang et al., 2013; Meric-Bernstam et al., 2017), leading to strong inhibitors. Tan and co-workers introduced the need for rational design to incorporate chemical staples by maintaining enthalpic interactions while reducing entropic costs (Tan et al., 2016). Our work on this field has centered on identifying bound conformations through integrative modeling approaches based on molecular dynamics simulations (Morrone et al., 2017a; Morrone et al., 2017b; Lang and Perez, 2021). We have been able to predict the binding of several linear and cyclic peptides as well as qualitative relative binding free energies (Morrone et al., 2017b). We identify different binding mechanisms for different peptides (Lang and Perez, 2021): p53 which is intrinsically disordered binds in a disordered state and then folds in the active site, whereas ATSP-7041 (**4** in Figure 3) is a stapled peptide that binds as a helix. In the latter case, an incorrect orientation of the side chains requires partial unbinding and rebinding of the stapled inhibitor. Due to long residence times, even partial unbinding can be a slow step in simulations, leading to slow convergence. A linear peptide (pdiq) with strong helical propensities is shown to be able to rearrange its side chains by partially unfolding in the active site. Thus, pdiq avoids the slow unbinding step.

## The BH3-Bcl-2 Interaction

Apoptosis is an evolutionarily conserved, regulated form of cell death involved in tissue homeostasis, embryonic development and immunity (Elmore, 2007). Apoptosis dysregulation has a major impact in disease, since an excessive response can lead to neurodegeneration or an increased ischemic risk, whereas a defective response plays a major role in tumor development and autoimmune diseases (Favaloro et al., 2012). The intrinsic apoptotic pathway (physiologically dominant and not mediated by a death receptor) is regulated through a complicated PPI network involving the B-cell lymphoma-2 (Bcl-2) family of proteins. With some of the members exhibiting pro-apoptotic activity and others pro-survival profiles, the apoptotic process is initiated by the interaction of pro-apoptotic and pro-survival members regulating mitochondrial outer membrane permeability, a crucial step in apoptosis (Chipuk et al., 2010; Czabotar et al., 2014). The interplay of the members of this family of proteins represents a good example illustrating how a short linear peptide motif is key to regulate a complex network of PPIs.

**FIGURE 4:**
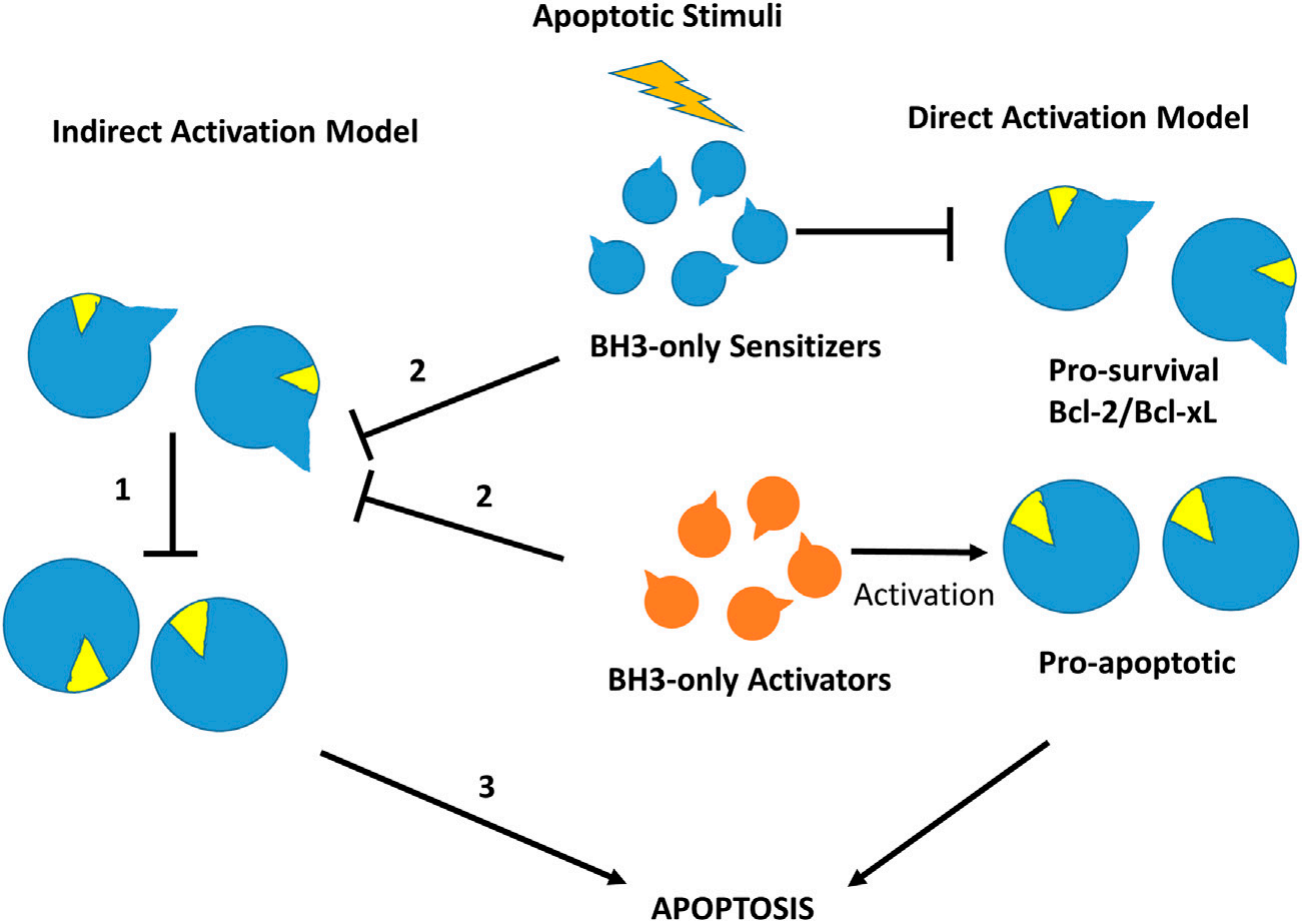
Direct and indirect mechanism models for Bax or Bak activation in the intrinsic cell death pathway. In the indirect activation model **(left)**, in the resting state effectors Bax and Bak are inhibited directly by pro-survival members (1). After an apoptotic stimulus, BH3-only proteins bind to pro-survival proteins releasing pre-activated Bak and Bax (2) to initiate the apoptosis process (3). In the direct activation model **(right)**, after an apoptotic stimulus BH3-only activators activate Bak/Bax, whereas BH3-sensitizers inhibit pro-survival Bcl-2 members, initiating the apoptotic process.

More than 20 members of the Bcl-2 protein family have been characterized so far. Sequence analysis indicates that they share one or more specific conserved regions known as Bcl-2 homology (BH) domains that are necessary for function, since their deletion via molecular cloning affects survival/apoptosis rates. Pro-survival members such as Bcl-2, Bcl-xL, Bcl-w, Mcl-1, Bfl1/A1 and Bcl-B are characterized for exhibiting four homology domains (BH1-BH4) together with a transmembrane domain. On the other hand, pro-apoptotic members can be classified into two subgroups: the multi-BH domain proteins including the pro-apoptotic effectors Bax and Bak with four BH domains (BH1-BH4) together to a transmembrane domain and the BH3-only proteins such as Bim, Bid, Puma, Bad, Bik, Bmf, Hrk and Noxa that share little sequence homology, apart from the BH3-domain (Chittenden, 2002). Some of the BH3-only proteins like Bim, Bid and to a lesser extend Puma are direct activators of the pro-apoptotic effector proteins, whereas the rest are sensitizers that indirectly activate Bak and Bax by binding to pro-survival proteins and liberating BH3-only activators (Singh et al., 2019) (Figure 4).

Despite the progress made in the last years, the mechanism of apoptosis regulation by the Bcl-2 family of proteins is not completely understood (Ichim and Tait, 2016). It is well established that the relative amounts of pro-apoptotic and pro-survival proteins in a cell, together with the capability of these proteins to form heterodimers, determine cell susceptibility to undergo apoptosis (Oltvai et al., 1993). Moreover, it is well established that pro-survival proteins inhibit apoptosis by directly binding to and sequestering their pro-apoptotic counterparts. This hypothesis was demonstrated when the BH3 domain of pro-apoptotic proteins showed capability to induce apoptosis in cell-free systems and HeLa cells (Holinger et al., 1999; Wang et al., 2000). Thus, under normal conditions in healthy cells, pro-survival Bcl-2 proteins sequester pro-apoptotic effectors as well as BH3-only proteins, preventing apoptosis. However, upon cytotoxic stress, the overwhelming number of BH3-only proteins produced activate pro-apoptotic effectors either by direct binding or through the liberation of restrained pro-apoptotic effectors by binding to pro-survival Bcl-2 proteins. This process originates the accumulation and activation of pro-apoptotic effectors with their subsequent oligomerization at the mitochondrial outer membrane, inducing its permeability (Chipuk et al., 2010; Singh et al., 2019). Moreover, the variable affinities exhibited by the diverse members of family for each other and their modulation when proteins are embedded in a membrane are also relevant. Consequently, BH3 domain peptide analogs and surrogates have been long considered appealing molecules for therapeutical intervention (Goldsmith et al., 2006), especially in cancer, since downregulation of apoptosis is considered a key step for the initiation and maintenance of the disease (Hanahan and Weinberg, 2000; Hanahan and Weinberg, 2011).

**FIGURE 5:**
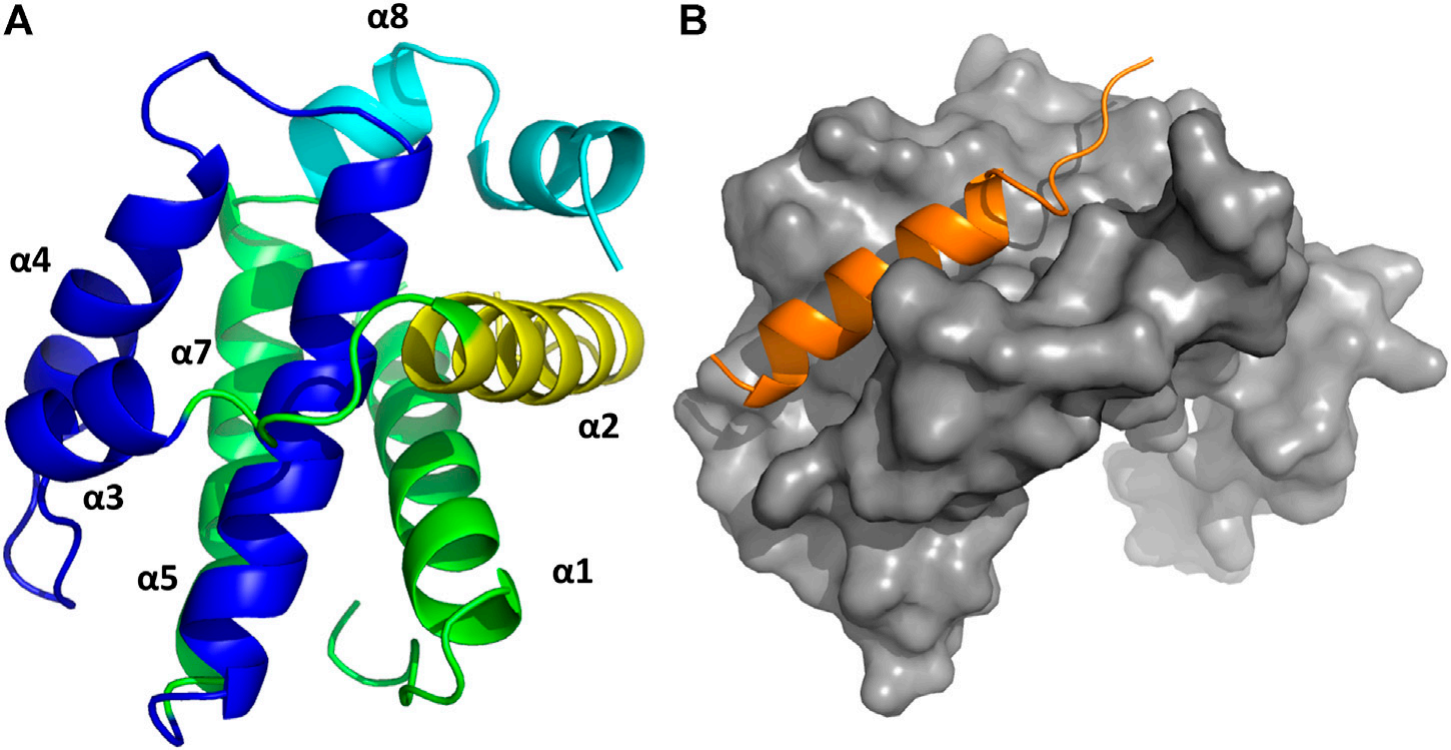
**(A)** 3D structure of Bcl-xL (PDB ID: 1MAZ) with the diverse elements of secondary structure labeled. The BH domains are color-coded: BH1 navy blue; BH2 cyan; BH3 yellow; BH4 dark green. **(B)** 3D structure of the complex Bcl-xL/BH3-Bak (in orange) (PDB ID:5FMK).

Structural studies on diverse members of the Bcl-2 family have provided a wealth of information, being key to understand the molecular mechanisms by which the intrinsic apoptosis pathway is regulated. The 3D structure of the human Bcl-xL in its apo form, solved by NMR spectroscopy and X-ray crystallography was the first one available (Muchmore et al., 1996). It consists of eight alpha helices (α1-α8) that are connected to the BH domains. Specifically, the BH2 spans along helix α8, the BH3 along helix α2 and the BH4 along helix α1. In contrast, the BH1 spans partially along helices α4 and α5 together to the loop connecting them (Figure 5A). In addition, the protein also has a C-terminal segment that serves as an anchor to the membrane, needed to be removed to conduct structural studies. A major feature of the structure is the large hydrophobic groove formed by the BH1-BH3 domains that corresponds to the interaction site of the BH3 domain of its counterpart family members, as shown in diverse crystallographic structures. The structures of the rest of the pro-survival members of the Bcl-2 family in their apo form exhibit the same topology (Petros et al., 2001; Hinds et al., 2003; Day et al., 2005; Harvey et al., 2018). Interestingly, the structures of the pro-apoptotic effectors Bak and Bax also exhibit the same topology, despite their diametrically opposing functions (Suzuki et al., 2000; Moldoveanu et al., 2006). In contrast, except for Bid that also exhibits a similar structure (Chou et al., 1999), most BH3-only proteins are intrinsically disordered (Hinds et al., 2007).

There are currently no heterodimer structures available involving Bcl-2 family members. However, several structures of pro-survival Bcl-2 members bound to BH3, the linear peptide motif involved in pro-survival-pro-apoptotic PPIs are available. The complex Bcl-xL-Bak BH3 domain was the first three dimensional structure solved of any Bcl-2 protein complex (Figure 5B) (Sattler et al., 1997). The structure shows the BH3 domain in a helical structure bound to a hydrophobic groove with four hydrophobic residues projecting their side chains into the cleft and with the Asp^83^ residue displaying an electrostatic interaction with Arg^139^ in Bcl-xL. Subsequently, several structures of BH3 domains bound to diverse pro-survival Bcl-2 members were reported in the literature, all of them exhibiting the same general features found in the Bcl-xL-Bak BH3 complex (Petros et al., 2004). Sequence alignment of the diverse BH3 domains shows they display the consensus sequence Ф_1_ΣXXФ_2_XXФ_3_ΣDZФ_4_Γ, where Ф_n_ represents a hydrophobic residue, Σ is a residue with a short side chain, Z an acidic residue, Γ is a hydrophilic residue and X represents any residue (Chen et al., 2005; Day et al., 2008; Lomonosova and Chinnadurai, 2008). The Ф_2_ residue is a leucine in all pro-apoptotic BH3-only members whereas Ile, Leu, Val, Met or an aromatic residue are frequently found for the rest Ф_n_. The pattern Ф_n_ display guarantees their alienation on the same face of a helical structure. These four residues together with the conserved aspartic acid are the residues responsible for binding the hot spots at the hydrophobic binding cleft consisting of four hydrophobic pockets (P1-P4) together with a conserved arginine. As mentioned above, the pro-apoptotic BH3 domains bind the pro-survival proteins with different affinities. Thus, Bim and Puma have comparable affinity for all pro-survival proteins. Bad and Bmf preferently bind Bcl-2, Bcl-xL and Bcl-w, whereas Noxa bind only Mcl-1 and Bfl1/A1 and Bid, Bik and Hrk bind Bcl-xL, Bcl-1 and Bfl1/A1 (Chen et al., 2005). This differential profile is due to differences in their sequence, since mutations can reverse binding preferences (Campbell et al., 2015). Numerous structural and computational studies have been performed to understand the nature of the binding preferences between BH3 peptides the diverse members of the Bcl-2 family, providing a deeper insight into the nature of these interactions key to design BH3 peptide surrogates and peptidomimetics (Lama and Sankararamakrishnan, 2011; Ivanov et al., 2016; Zhang et al., 2018; Vila-Julià et al., 2020). This knowledge is specially relevant for designing selective BH3 analogs (Denis et al., 2020; Reddy et al., 2020).

In order to design drugs with BH3 functionality, peptide analogs and surrogates are expected to be more resistant to proteolytic enzymes and can also be designed to exhibit higher populations of the bioactive conformation in solution compared to the original linear peptides. Diverse strategies have been used for the discovery of potent peptide surrogates of the BH3 domain (Orzáez et al., 2009). As a general strategy, increased resistance to proteolytic enzymes can be carried out by replacing peptide bonds by isosteres or using a holistic approach, constructing retro-inverso analogs or peptoids (Perez et al., 2002; Akram et al., 2014; Perez, 2018). The design of α/β peptide surrogates of BH3 with sequences alternating α and β amino acids have been successfully designed (Horne et al., 2008). Interestingly, the α/β alternating pattern can modify the pharmacodynamic profile of the analogs changing their affinity or moreover, their selectivity for the different pro-survival proteins (Boersma et al., 2012).

**FIGURE 6:**
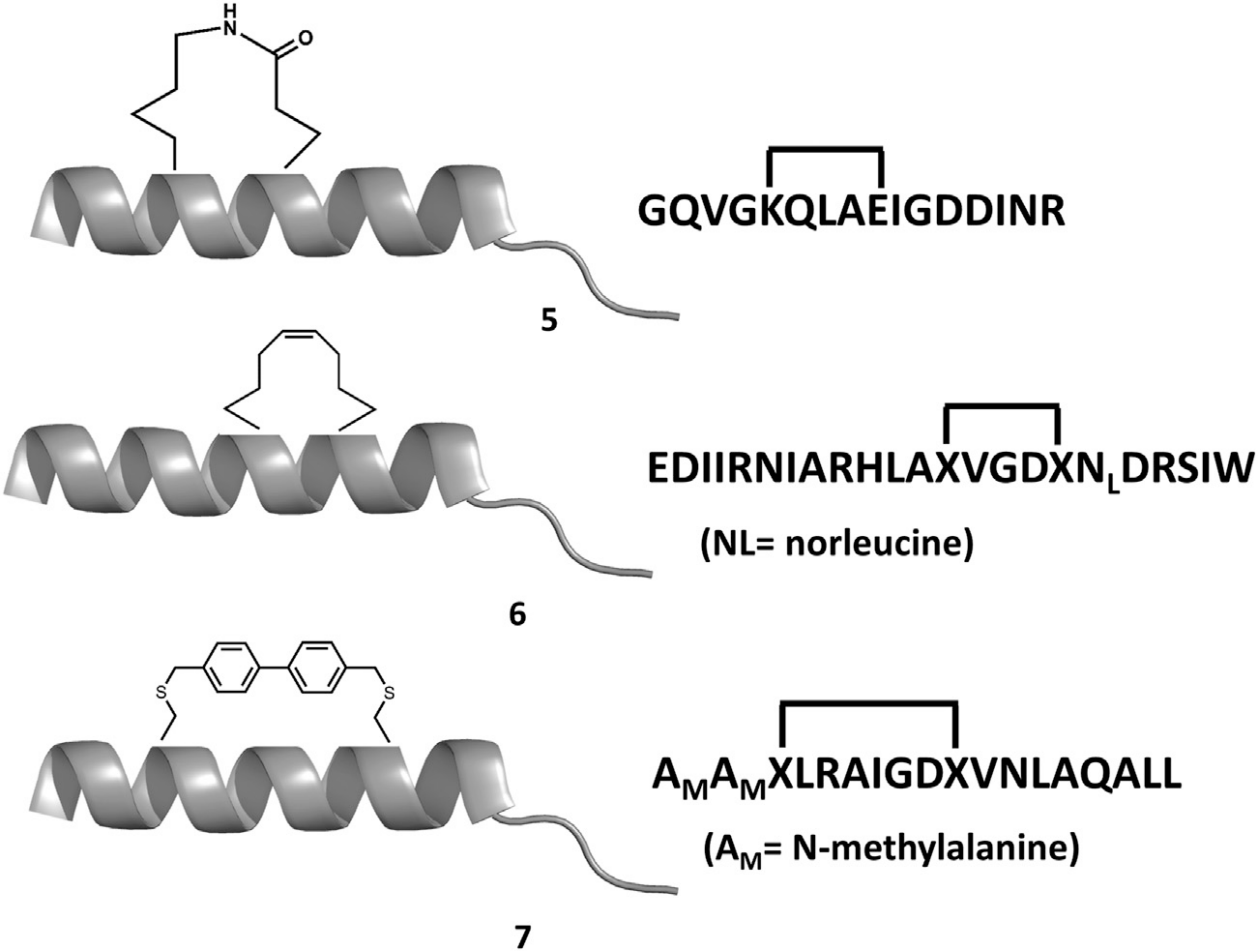
Chemical structures of diverse stapled analogs of the BH3 domain.

Experimental studies as well as computational studies reveal that BH3 peptides do not exhibit a helical structure in solution (Petros et al., 2000; Perez et al., 2016). In order to avoid negative configurational entropy effects, the affinity of the analogs can be increased by designing analogs that enhance the bioactive conformation population in solution by embedding helix enhancer residues in the sequence (Delgado-Soler et al., 2013). An alternative approach focused on constraining the helical structure of the analogs in solution. Early attempts to stabilize the Bak BH3 peptide used lactam cross-links side chain-to-side chain at positions i and i+4 (**5** in Figure 6). Unfortunately, although the peptides exhibit helical structure, none of these analogs showed any binding to Bcl-2 due to a steric hindrance with the receptor (Yang et al., 2004). In contrast, the hydrocarbon stapling approach demonstrated to be a successful approach. In this case, macrocyclization between specific residues of the helix occurs via a ring-closing metathesis reaction using α,α-di-substituted amino acids with olefin tethers as building blocks. This procedure was successfully used to stabilize the Bid BH3 peptide (**6** in Figure 6), proving to be helical, protease-resistant, and cell-permeable molecules that bound with increased affinity to multidomain Bcl-2 member pockets (Walensky et al., 2004). However, not every stapled BH3 helix exhibits improved bioactivity, which requires the synthesis and testing of a set of modified peptides to identify suitable candidates. Other types of staples have also been used. For example, bisaryl cross-linkers have been used recently to reinforce peptide helices containing two cysteines at positions i and i+7. This approach has been used for the stabilization of the Noxa BH3 peptide (**7** in Figure 6), showing a potent cell-killing activity in Mcl-1-overexpressing cancer cells (Muppidi et al., 2012). After an optimization process, the final molecule exhibits increased helicity in regard to the native peptide and an improved pharmacokinetic profile including cell permeability and proteolytic stability.

**FIGURE 7:**
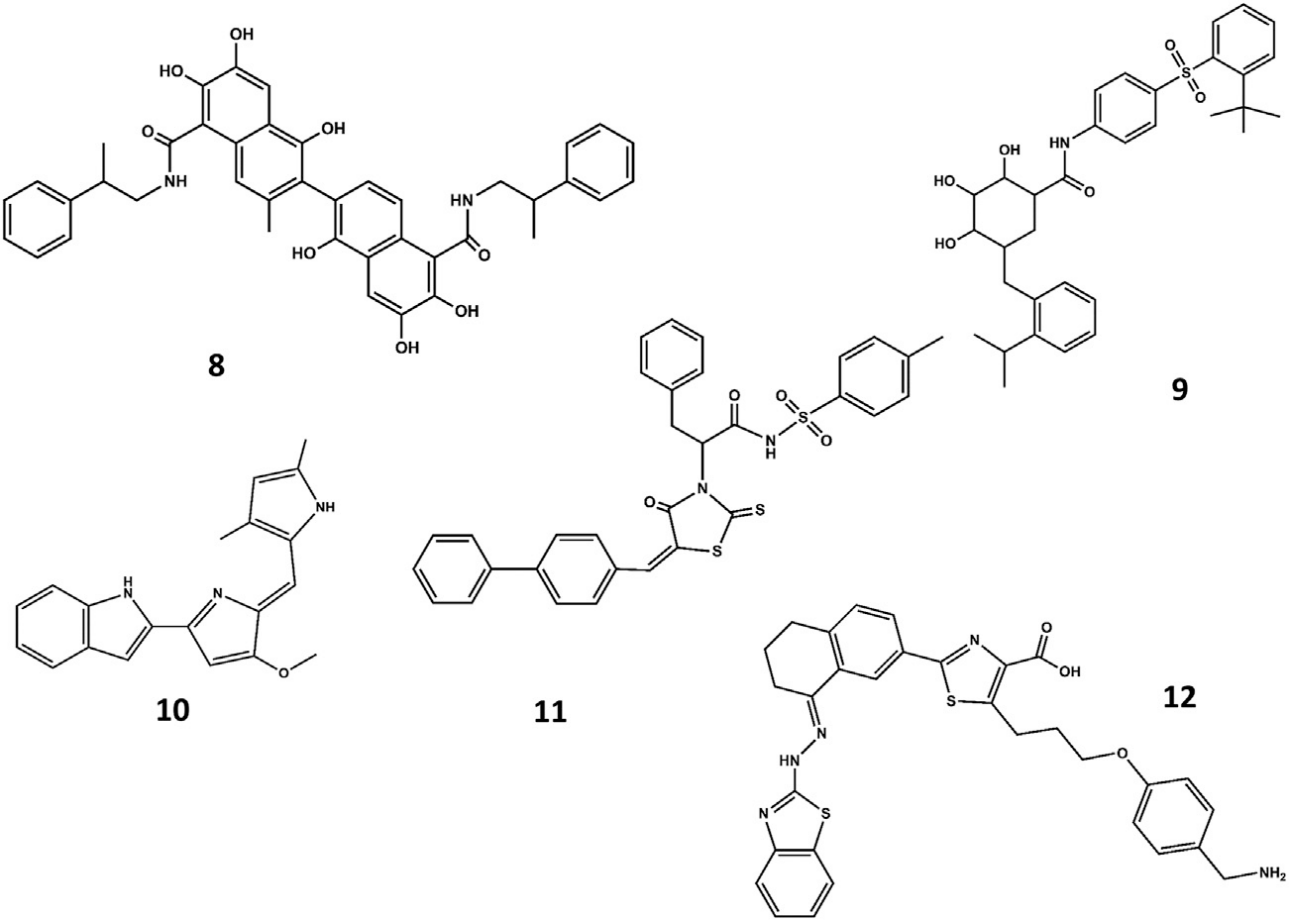
Chemical structures of diverse small molecule mimics of the BH3 domain. Sabutoclax (**8**); TW-37 (**9**); obatoclax (**10**); WL-276 (**11**); WEHI-539 (**12**).

A different strategy focuses on finding small-molecule mimics of the BH3 domains (Roy et al., 2014; Yap et al., 2017). Despite a-helix mimics have been reported (Yap et al., 2017), most of the BH3 mimetics have been discovered from an optimization process that started with a hit identified by high throughput screening, followed a hit-to-lead structure-based optimization process helped with biophysical techniques and computational methods. Hits identified from natural products libraries include antimycin A3 (Tzung et al., 2001), gossypol and purpurogallin (Kitada et al., 2003), epigallocatechin gallate and theaflavinin or the alkaloid prodigiosin (Chang et al., 2011). On the other hand, hits found in commercial libraries include BH3I-1 and BH3I-2 (Degterev et al., 2001). Gossypol was used as starting structure to discover interesting Bcl-2 inhibitors (Becattini et al., 2004) like sabutoclax (**8** in Figure 7) (Wei et al., 2010) or TW-37 (**9** in Figure 7) (Wang et al., 2006). Similarly, prodigiosin was used as starting molecule to discover obatoclax (**10** in Figure 7), an inhibitor of pro-survival members of the Bcl-2 family that antagonize Bax or Bak, causing cytotoxicity. The compound has gone through several clinical II studies for the treatment of patients with solid tumors and hematopoietic malignancies (Shoemaker et al., 2006). On the other hand, optimization of BH3I-1 led to the discovery of WL-276 (**11** in Figure 7) (Wang et al., 2008) with a similar inhibitory activity against Bcl-2 and enhanced inhibitory activity against Bcl-X_L_ as compared with gossypol. In contrast, WL-276 effectively induces apoptosis in PC-3 cells at low micromolar concentrations. Similarly, the compound WEHI-539 (**12** in Figure 7) was optimized from a hit identified in high throughput screening following a structure-guided approach. The compound exhibits high affinity and selectivity for BCL-X_L_ and potently kills cells by selectively antagonizing its pro-survival activity (Lessene et al., 2013).

**FIGURE 8:**
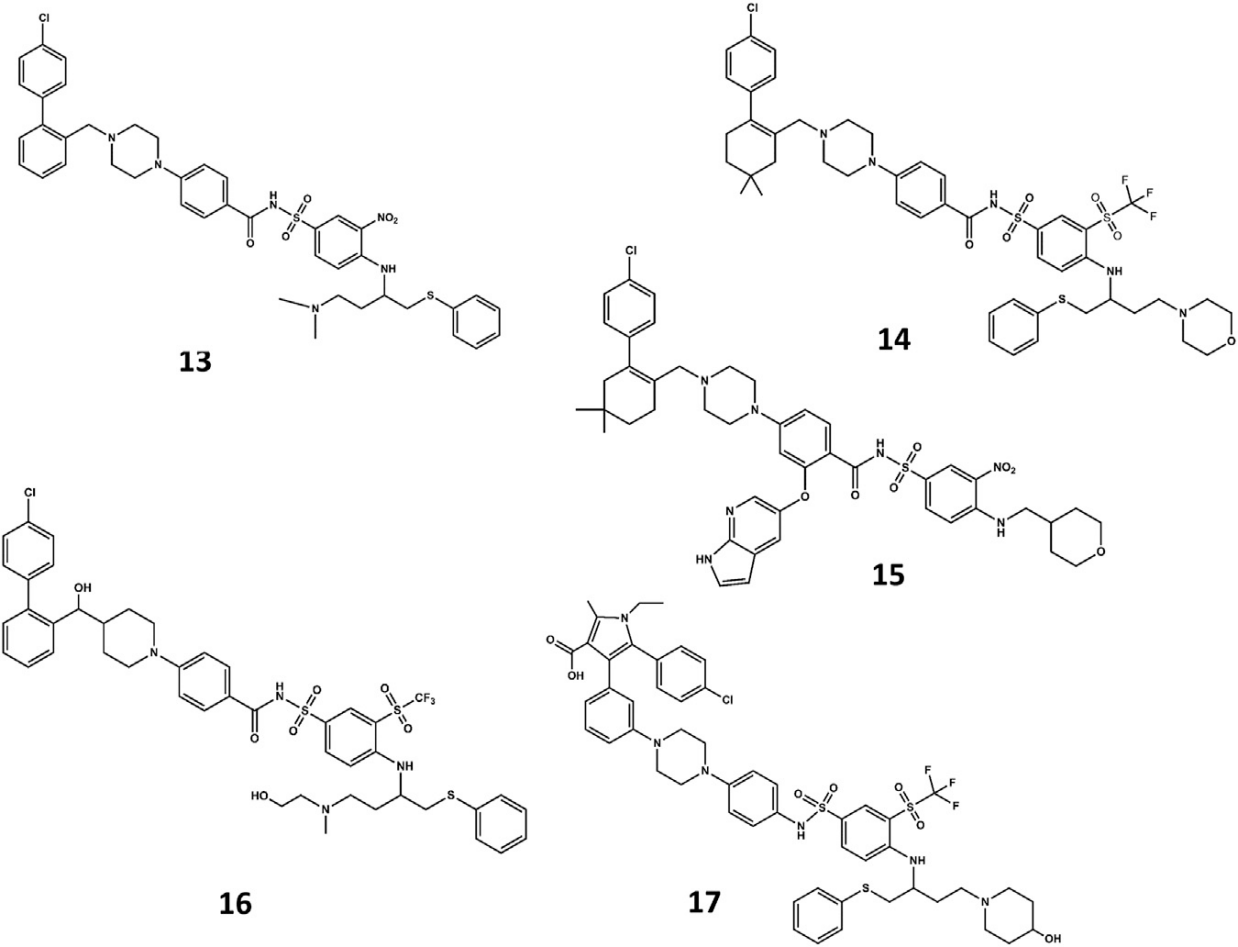
Chemical structures of diverse small molecule mimics of the BH3 domain. ABT-737 (**13**); Navitoclax (ABT-263) (**14**); venetoclax (ABT-199) (**15**); BM-975 (**16**); AZD4320 (**17**).

**FIGURE 9:**
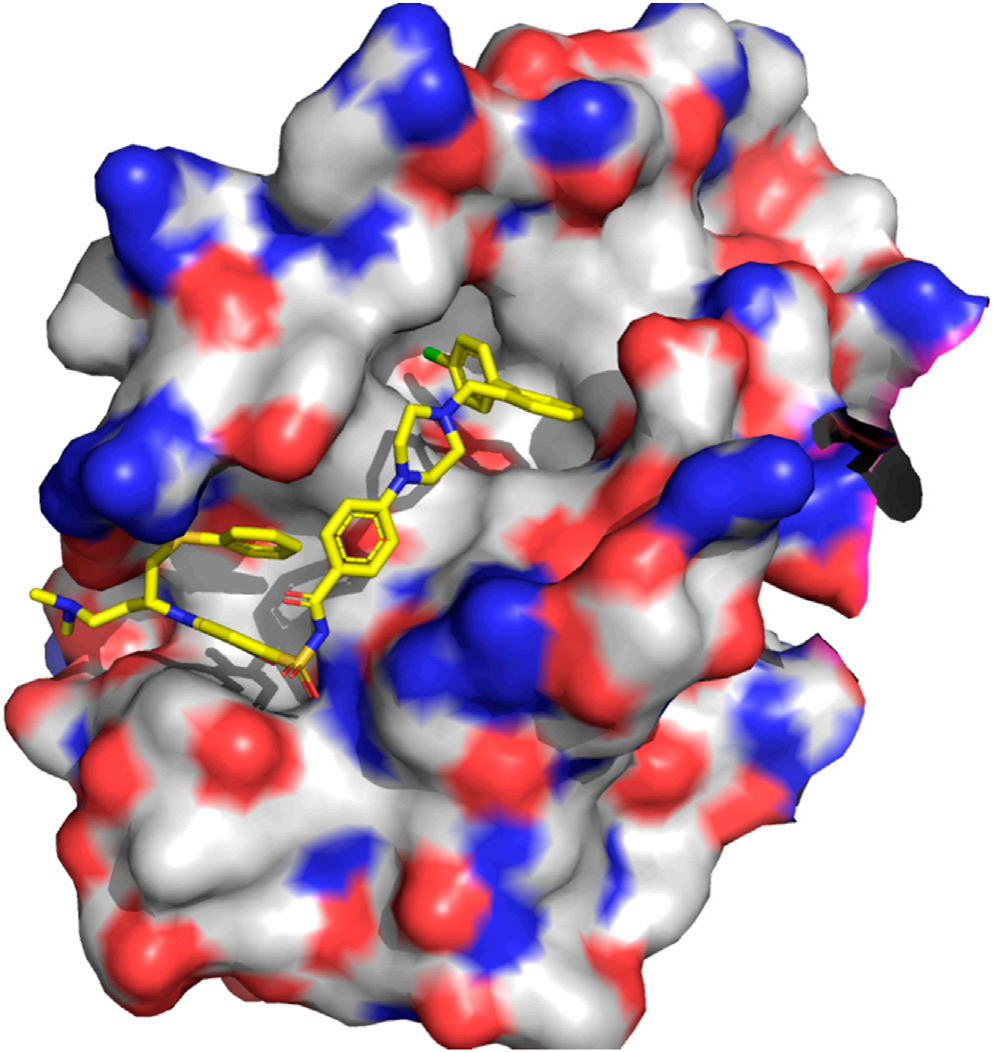
3D structure of the complex Bcl-xL/ABT-737 (PDB ID: 5MFK).

A NMR spectroscopy based fragment screening approach coupled with computational studies was also used to discover other small-molecule inhibitors of Bcl-2. Specifically, these studies led to the discovery of ABT- 737 (**13** in Figure 8) (Oltersdof et al., 2005), a potent small-molecule inhibitor of Bcl-2, Bcl-xL, and Bcl-w. Although the compound is not orally available, it exhibits an acceptable pharmacokinetic profile when administered intraperitoneally. The crystallographic structure of the complex with Bcl-x_L_ (Lee et al., 2007) shows ABT-737 sitting on the hydrophobic BH3 binding cleft as expected from its design (Figure 9). Further optimization of the molecule led to the discovery of the orally bioavailable Navitoclax (ABT-263) (**14** in Figure 8) (Tse et al., 2008). Both compounds are peptidomimetics of the Bad-BH3 domain, so they are potent binders of Bcl-2, Bcl-xL and Bcl-w, but not to Mcl-1 or Bfl1/A1, exhibiting a demonstrated antitumor activity *in vitro* and *in vivo*. While clinical responses with navitoclax were promising, mechanistic dose-limiting thrombocytopoenia was observed in patients under treatment due to Bcl-xL inhibition in platelets. Further studies were undertaken to remove the undesired side effects, leading in the discovery of venetoclax (ABT-199) (**15** in Figure 8) (Pan et al., 2014), a highly selective Bcl-2 inhibitor that was approved by the US FDA in April 2016 as a second-line treatment for chronic lymphocytic leukemia. Inspired by ABT-737, a series of inhibitors with a 4,5-diphenyl-1H-pyrrole-3-carboxylic acid as core structure have been developed. Among them, compound BM-957 (**16** in Figure 8) that binds to Bcl-2 and Bcl-xL with high affinity and shows potent activity in cell growth inhibition in small-cell lung cancer cell lines like the H1147 and H146 (Chen et al., 2012). Also inspired by ABT-737 and following a structure based process, AZD4320 (**17** in Figure 8) was recently developed (Balachander et al., 2020). This is a dual inhibitor of Bcl-2 and Bcl-xL that minimizes Bcl-xL–mediated thrombocytopenia. The compound has been used satisfactorily to design AZD0466, a drug-dendrimer conjugate, where AZD4320 is chemically conjugated to a PEGylated poly-lysine dendrimer (Patterson et al., 2021).

## Integrins and Tissue Engineering

Integrins orchestrate cell–cell and cell–extracellular matrix adhesive interactions from embryonic development to mature tissue function involving PPIs with other proteins located in the extracellular matrix (ECM) of tissues (Barczyk et al., 2010). The ECM refers to different molecules that are secreted by cells, mainly polysaccharides, small molecules and proteins, that serve as matrix that provide structural and biochemical support to the surrounding cells (Frantz et al., 2010). Such interactions occur naturally in native tissues and can be altered in the presence of injuries or damaged tissues. In this context, and in order to allow tissue regeneration, the discipline of tissue engineering has emerged in order to guide cells, tissues and ultimately, organs, to recover their function (Langer and Vacanti, 1993). However, integrins not only play an important role in regulating cell adhesion, but also function as integral transmembrane signaling molecules in the regulation of cellular behavior such as organization of the intracellular cytoskeleton, regulation of the cell cycle or movement of new receptors to the cell membrane (Giancotti and Ruoslahti, 1999; Kadry and Calderwood, 2020). They are also involved in blood coagulation and in pathophysiological processes such as tumor growth, metastasis, or angiogenesis. Specific functions of integrins have been exploited for therapeutical intervention. Thus, diverse PPIs disruptors have been designed for the treatment of thrombosis, stroke and myocardial ischemia (Scarborough and Gretler, 2000) or for the treatment of cancer and osteoporosis (Auzzas et al., 2010). On the other hand, knowledge of the nature of the PPIs involved with the ECM can be used to mimic integrin ligands in tissue engineering.

Integrins are heterodimers integral transmembrane proteins composed of α and β subunits that modulate the association between the extracellular matrix and the cytoskeleton. More than twenty different α/β heterodimeric integrins have been recognized, resulting from the combination of eighteen α and eight β subunits (Hynes 2002; Barczyk et al., 2010). The most widely studied members of this subgroup have in common a β3 subunit. Specifically, the platelet receptor αIIbβ3 that binds fibrinogen, involved in the blood coagulation process and, the fibronectin receptor αvβ3 that binds a wide variety of ligands and is up-regulated in many solid tumors contributing to the mechanisms involved in tumor growth and metastatic dissemination. Integrins signaling is initiated by binding to different proteins in the ECM including fibronectin, laminin, vitronectin, collagen or cell-surface receptors such as intercellular adhesion molecule-1 (ICAM-1) and vascular cell-adhesion molecule-1 (VCAM-1).

Integrins and ECM proteins PPIs occur via short peptide linear binding motifs (Ruoslahti, 1996). One of these motifs is the sequence Arg-Gly-Asp (RGD), common to a subgroup of eight integrins that was the object of study for designing inhibitors of platelet aggregation. The RGD motif is not a potent inhibitor of αIIbβ3 in platelet aggregation assays. However, extension with an amino acid at the C-terminal significantly enhances the inhibitory activity of these peptides (Pierschbacher and Ruoslahti, 1984). Subsequent studies by means of NMR conformational analysis and molecular dynamics simulations led to the design of cyclic peptides embedding the RGD sequence. These studies demonstrated that analog selectivity for different integrins was associated with the conformation the RGD segment attains. Specifically, the distance between the basic arginine side chain and the acid aspartic side chain controlled by the conformation of the macrocycle. In the αIIbβ3 selective pentacyclic peptides the peptide adopts a conformation with a β-turn centered on Gly together with a distorted type II′ β-turn involving the two other residues at the i+1 and i+2 positions, respectively (Müller et al., 1994). On the other hand, pentacyclic peptides selective for the αvβ3 receptor exhibit a reverse β-turn centered on the Asp residue and a distorted type II′-β turn with Gly and Asp at the i+1 and i+2 positions, respectively. In the case of the hexacyclic peptides, selectivity could be shifted from αIIbβ3 to αvβ3 by forcing the conformation attained by the peptide from a type II′ to a type I β-turn conformation. This forces the arginine side chain either to adopt a pseudoequatorial orientation or to be raised above the plane of the backbone in a pseudoaxial orientation, respectively (Bach et al., 1996). These studies led to the discovery of selective antagonists of the αIIbβ3 and αvβ3 receptors (Andronati et al., 2004; Auzzas et al., 2010) including the commercially available cyclic heptapeptide eptifibatide, a potent αIIbβ3 selective antagonist (Scarborough et al., 1993), and the cyclic pentapeptide cilengitide, a potent αvβ3 selective antagonist (Dechantsreiter et al., 1999).

The concept of tissue engineering aims at stimulating the regeneration of a damaged tissue (Perez et al., 2015). For this purpose, the combination of three main elements is essential in order to provide the appropriate signaling to induce the desired effect (Perez et al., 2013). The three key elements are: scaffolds, short linear peptide motifs and cells. The aim of these three main components is to mimic to the maximum extent the situation that takes place in natural tissue healing (Perez et al., 2015). In this sense, the scaffold serves as a matrix that presents similar characteristics to the natural extracellular matrix of tissues. The scaffold can be loaded with molecules involved in the regeneration of the tissues, which depending on the tissues, can involve growth factors, cytokines or drugs (Garg et al., 2012). Finally, these constructs will allow the attachment, proliferation and differentiation of cells that will ultimately, once implanted in the site of defect, guide the surrounding tissues to induce new tissue formation. Among the three different elements, scaffolds play a pivotal role in providing the appropriate cues to allow the interplay between cells and tissue with the scaffolds itself (Perez et al., 2013).

Scaffolds can be composed of different types of materials, which can be from polymeric origin, ceramics or metallic (O’Brien, 2011). The selection of the materials will mainly depend on the targeted tissue to be regenerated, using soft materials (polymers) for soft tissues such as neuronal tissues and cartilage, and stiff materials (ceramics and metals) for hard tissues, mainly bone (Engler et al., 2006). In all cases, these scaffolds need to mimic to some extent the ECM. In order to replicate the attachment of cells onto the ECM, synthetic materials need to provide similar ECM cues to allow the protein-protein interaction that will eventually lead to cell attachment and guide cells into the regeneration processes (Perez et al., 2013). Hence, the scaffolds, if properly selected, can act as mediator and guidance for the regeneration of tissues. For this purpose, the scaffolds themselves can be composed of natural proteins which possess cell recognition sites (epitopes) that will allow the interaction with cells, or they can be designed as a non-protein origin (Dhandayuthapani et al., 2011). In the latter case, it is essential to provide within the scaffolds certain cues to allow cell-material interaction (Tallawi et al., 2015). For this purpose, several domains can be covalently attached on the surface of the materials, such as proteins, peptides, drugs or growth factors to name a few. Generally speaking, proteins present certain cell instructive domains that will allow the recognition by cells and allow interaction. Nevertheless, the presence of proteins within the body can induce non-desired effects such as an acute immune response. Furthermore, as proteins are big molecules, their epitopes need to be properly exposed for cells to interact, otherwise their positive effect can be neglected. Taking this into account, peptides have appeared as domains that can be used as well, allowing only using the specific amino acid sequence that is active for cell adhesion or cell guidance with the added value that will prevent from any immune response and will be easily allocated in the proper manner to allow cell interaction (Perez et al., 2013; Tallawi et al., 2015; Klimek and Ginalska, 2020).

Regarding the short linear peptide motifs, there have been a number of epitopes discovered to have relevant properties in specific cell functionalities. The most widely known amino acid sequence is RGD (Arg-Gly-Asp), which is an amino acid sequence found in several ECM proteins, such as collagen, laminin, fibronectin or vitronectin, mainly having key role in cell adhesion (Jeschke et al., 2002; Petrie et al., 2008; Von Der Mark et al., 2010). Several peptides possess similar potential to allow cell adhesion, such as PHSRN and YIGSR (Fittkau et al., 2005). There are other amino acid sequences with different potential and with more specific targets. For instance, IKVAV, which is an epitope from the α-1 laminin chain, enhances cell growth, neuronal differentiation and nerve regeneration (Patel et al., 2019). In a similar way, KLPGWSG induces as well neuronal differentiation (Caprini et al., 2013). While these peptides are related with ECM proteins, other peptides have mimicked specific growth factors, such as bone morphogenetic protein, vascular endothelial growth factors or brain derived neurotrophic factors. These growth factors are molecules that are released from cells into the ECM and have great therapeutic potential. In this sense, peptide such as KPSS, KLT and RGI are able to regulate bone regeneration, angiogenesis and nerve regeneration respectively (Ding et al., 2020).

Despite general knowledge on how certain epitopes are able to guide cell behavior, there are still undiscovered number of epitopes that could provide similar potential to those already known. While these epitopes could be empirically and experimentally discovered, this would be both time consuming and not cost-effective. For this reason, computational strategies open a window of unlimited epitope discovery that will more finally tune tissue guidance. Up to now, sequences are not taken into account as cell specific, or in other words, as integrins specific, since not all cells present that same cell membrane integrins. Discovering among the selective interactions of certain integrins with specific peptides may be of great potential.

## Opportunities for Computational Approaches in Tissue Engineering

A straightforward application of the principles described in the cases of p53 and bcl2 into tissue engineering is the identification of naturally occuring binding modes and interactions between ECM elments and integrins. This knowledge can lead to the identification of functional peptide motifs that have not currently been identified and the design of new chemical molecules that bind certain integrins specifically. As an example, the RGD motif has been characterized using computational and experimental tools due to its ability to bind different integrin types. However, the interplay of internal structure of the motif and binding mode give rise to nuances in the binding and recognition (Dechantsreiter et al., 1999; Kapp et al., 2017). Thus, the presence of an RGD motif inside a particular scaffold might force conformations of the motif that are only recognized by a subset of integins. A hybrid computational and experimental approach used metadynamics based sampling to design a small molecule antagonist (RUC-1) that is specific to only one type of integrin (Zhu et al., 2010). The native RGD antagonist binds different integrins and induces a conformational change between a closed and an open state; whereas the RUC-1 antagonist binds only in the αIIbβ3 integrin without inducing a change between the closed and open state (Zhu et al., 2010). Other computational approaches target membrane proteins like integrins by optimizing the sequence of the peptide epitope that binds the membrane receptors (Yin et al., 2007; Shandler et al., 2011).

**FIGURE 10:**
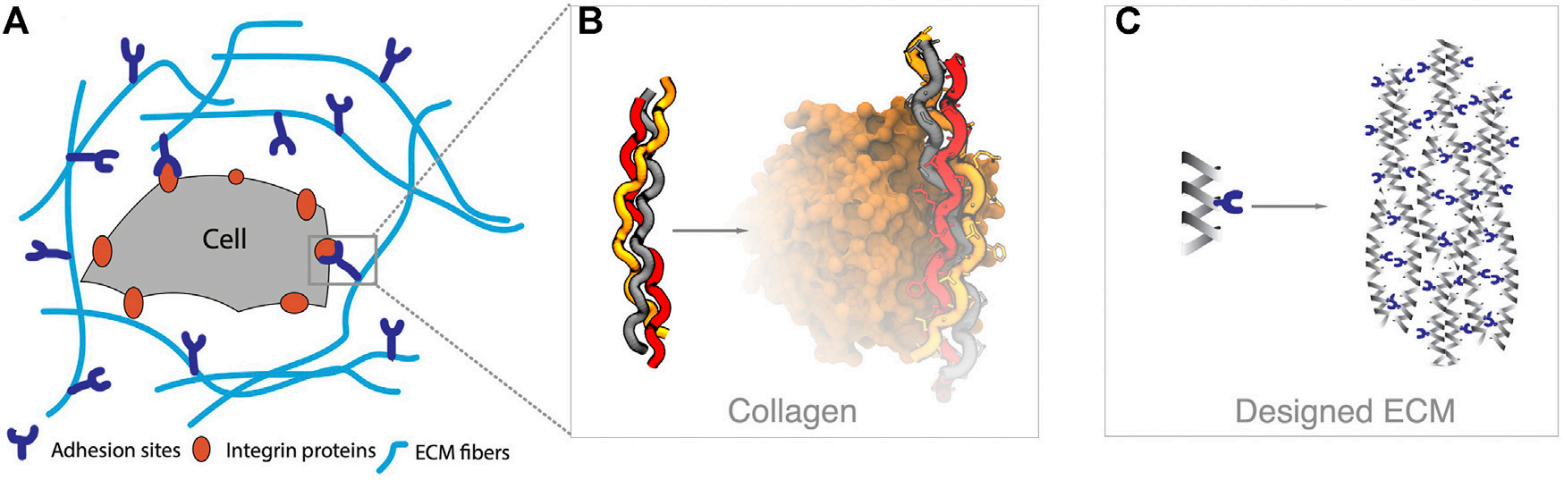
Tissue engineering overview. **(A)** The surface of a cell is covered with different types of integrin proteins, which recognize molecular queues in the extracellular matrix (ECM) environment. **(B)** The ECM is composed of diverse biological components, such as collagen fibers, which can interact with integrins through specific amino acid sequences (e.g., GFOGER in the example, pdbid 1dzi). **(C)** The goal in tissue engineering is to design materials, such as self-assembling peptides that provide an ECM scaffold and chemical queues that can trigger cellular behavior.

A second area of interest is in the development of material that self-assembles to provide a scaffold for cells. A common strategy is to use self-assembling peptides that can from hydrogels on injection into a patient–thus localizing to a specific region in the body (Wade et al., 2012; Loo et al., 2015). There are several approaches to achieve molecular self-assembly which have been previously described experimentally (Loo et al., 2015), many of which rely on peptide sequences. These self-assembling peptides should have a sequence capable of self-assembling, while at the same time carrying a sequence motif that is accessible and recognizable by integrins, to dictate their behavior (see Figure 10). This is a chance for computational approaches to carry out systematic analysis of optimal sequence design to achieve specific mechanical properties (e.g., mechanical stiffness), and rational optimization for active and accessible conformations of the integrin-binding motifs. Smadbeck and co-workers designed short peptide motifs that were able to self-assemble and did a posterior characterization using experimental approaches (Smadbeck et al., 2014), a second strategy is based on using proteins that fold into stable structures and then self-assemble (Chen et al., 2019). Despite these successes in computational design, many hybrid approaches rely on an experimental design followed by computational characterization, typically with molecular dynamics (Cormier et al., 2013; Leonard et al., 2013). The accumulated knowledge on peptides secondary structure propensities (Jones, 1999; Moreau et al., 2009; Best et al., 2012), protein structure and assembly prediction (Lensink et al., 2007; Moult et al., 1995) and design principles (Simons et al., 1999; Delgado et al., 2019) can provide useful insights in this area of research.

## Conclusion

Computational and modeling approaches have long been involved in rational drug discovery. As the methods have matured, new opportunities emerge to model other types of molecules that can be beneficial as drugs, such as peptides. The challenges for computational methods continue to be related to sampling, scoring and the nature of the system under study. In systems where the protein receptor and ligand are rigid, predicting bound conformations is less challenging, and the quality of the scoring function dictates the accuracy of the predictions. As the systems become more flexible (both protein and ligand) requiring large conformational transitions and ability to bind through several possible binding modes, the interplay between sampling and scoring becomes more relevant to account not only for direct interactions but the ability to adopt bound conformations. General strategies for design include maximizing peptide interactions in the bound conformation and deriving peptidomimetic or small molecule solutions that maintain the interaction profile while reducing the internal flexibility of the ligand. We have highlighted opportunities to apply these methods to the field of tissue engineering, where recent advances in structure prediction can play a big role.
